# Colorectal cancer diagnostic model utilizing metagenomic and metabolomic data of stool microbial extracellular vesicles

**DOI:** 10.1038/s41598-020-59529-8

**Published:** 2020-02-18

**Authors:** Da Jung Kim, Jinho Yang, Hochan Seo, Won Hee Lee, Dong Ho Lee, Sungmin Kym, Young Soo Park, Jae Gyu Kim, In-Jin Jang, Yoon-Keun Kim, Joo-Youn Cho

**Affiliations:** 1 Department of Clinical Pharmacology and Therapeutics, Seoul National University College of Medicine and Hospital, 101 Daehak-ro, Jongno-gu, Seoul, 03080 Republic of Korea; 2 Institute of MD Healthcare Inc, Seoul, Korea; 3 Department of Internal Medicine, Seoul National University Bundang Hospital, Gyeonggi-do, Republic of Korea; 4 Department of Internal Medicine, Inje University Haeundae Paik Hospital, Inje University College of Medicine, Busan, Republic of Korea; 5 Department of Internal Medicine, Chung-Ang University College of Medicine, Seoul, Republic of Korea; 6 Department of Health and Safety Convergence Science, Korea University, Seoul, Republic of Korea; 7 Department of Biomedical Sciences, Seoul National University College of Medicine, 101 Daehak-ro, Jongno-gu, Seoul, 03080 Republic of Korea

**Keywords:** Metabolomics, Microbiology techniques, Colon cancer

## Abstract

Colorectal cancer (CRC) is the most common type cancers in the world. CRC occurs sporadically in the majority of cases, indicating the predominant cause of the disease are environmental factors. Diet-induced changes in gut-microbiome are recently supposed to contribute on epidemics of CRC. This study was aimed to investigate the association of metagenomics and metabolomics in gut extracellular vesicles (EVs) of CRC and healthy subjects. A total of 40 healthy volunteers and 32 patients with CRC were enrolled in this study. Metagenomic profiling by sequencing 16 S rDNA was performed for assessing microbial codiversity. We explored the small molecule metabolites using gas chromatography-time-of-flight mass spectrometry. In total, stool EVs were prepared from 40 healthy volunteers and 32 patients with CRC. Metagenomic profiling demonstrated that bacterial phyla, particularly of *Firmicutes* and *Proteobacteria*, were significantly altered in patients with colorectal cancer. Through metabolomics profiling, we determined seven amino acids, four carboxylic acids, and four fatty acids; including short-chain to long chain fatty acids that altered in the disease group. Binary logistic regression was further tested to evaluate the diagnostic performance. In summary, the present findings suggest that gut flora dysbiosis may result in alternation of amino acid metabolism, which may be correlated with the pathogenesis of CRC.

## Introduction

Colorectal cancer (CRC) is the most common type cancers in the world^1^. The majority of CRC occurs sporadically, indicating that environmental influences are the predominant cause of the disease^2^. Dietary pattern has long been considered as the most important lifestyle risk factor for CRC. *In vivo* and *in vitro* studies have investigated the effect of protein intake on CRC risk and suggest that consumption of excessive protein could lead to DNA damage and influence on the maintenance of colonocyte intergrity^3,4^. Diet-induced changes in gut-microbiome are recently supposed to contribute on epidemics of CRC. Accordingly, studies have suggested that the intestinal microbiome might be important for CRC initiation and progression, since tumors preferentially develop in the distal colon and rectum, which are colonized by approximately 70% of host microbiomes^2,5^. The microbiome has the potential to generate a microenvironment that favors the development of CRC, presumably by recruiting mediators such as interleukins, tumor necrosis factor-alpha, and reactive oxygen species^6,7^. Furthermore, metabolic products of the gut microbiota might increase the risk of developing colorectal cancer. For example, high levels of acetaldehyde produced by the gut microbiota can break down colonial folate, thereby increasing CRC risk^7^.

Microbe-derived extracellular vesicles (EVs) are emerging as an important new research subject in understanding the intersection of the gut-microbial communities and human health. Gut microbiota can secrete different types of EVs, including outer membrane vesicles (OMVs), shedding vesicles, and apoptotic bodies^8,9^. EVs are mainly composed of lipids, proteins, nucleic acids, and metabolites^10–12^. Although the underlying mechanisms are still unclear, their primary role is to transport active biomolecules into cells over long distances, providing drug delivery to target sites or regulating host cellular responses^11,13^.

Recent studies have provided mechanistic evidences for the participation of the gut flora in CRC development. An *in vivo* study demonstrated that genetically engineered animal model of CRC develop fewer tumors under germ-free conditions compared to those with a conventional microbiota^12^. Further, *Enterococcus faecalis* and *Escherichia coli* produce extracellular genotoxins and free radicals targeting DNA that can contribute to CRC development^14^. However, it is not yet clear which disease-causing signals are produced by bacteria in the gut. In this study, we profiled the microbiome and metabolites within EVs from CRC patients and healthy controls using 16 S ribosomal DNA (rDNA) amplicon sequencing and global metabolomics, respectively, to develop diagnostic models to assess the risk of CRC.

## Materials and Methods

### Research subjects

A total of 32 patients with colorectal cancer from Seoul National University Bundang Hospital and Chung-Ang University Hospital and 40 healthy control individuals from Haewoondae Baek Hospital participated in the present study between April 2016 and April 2018. All patients with colorectal cancer were diagnosed for the first time according to the diagnostic criteria proposed by the International Union Against Cancer and the American Joint Committee on Cancer in 2013^15^. The patients characteristics, such as age, sex, stage, tumor location, and carcinoembryonic antigen (CEA) test, were examined. Healthy subjects recruited for this study visited the hospital for a regular health screening. After the checkup, we selected healthy controls who were confirmed to have no known diseases and normal laboratory test results. The exclusion criteria for healthy controls included gut disease diagnosis, taking medication for gut disease, and previous CRC diagnosis. For healthy control individuals, general characteristics were recorded, including age, sex, and medical history. Patient and healthy subject exclusion criteria included colorectal cancer recurrence post-surgery, chemotherapy, complication of colorectal cancer with any other cancers or metabolic diseases, medication, or antibiotic treatment within 1 month of sample collection. Characteristics of subjects are shown in Table S1. The present study was approved by the Institutional Review Board of Seoul National University Bundang Hospital (IRB No. B-1708/412-301) and Haewoondae Baek Hospital (IRB No. 129792-2015-064), and was conducted in accordance with the principles of the Declaration of Helsinki. Informed consent was obtained from all subjects.

### Sample collection and EV isolation

Stool samples were collected prior to surgery or bowel preparation. All participants consumed a bland diet and did not smoke or consume alcohol 1 day prior to sample collection. A stool sample was collected from the center of the stool using a sterilized cotton swab and stored at −20 °C. Detailed procedure of sample collection was followed a previous study^16^. Prior to separation of bacterial EVs from stool, a stool sample (1 g) was mixed with 10 mL of phosphate-buffered saline (PBS) followed by vibration for 24 h. The samples were then incubated to separate the EVs from human stool; EVs from the stool samples were then isolated using centrifugation at 10,000 × *g* for 10 min at 4 °C. Bacteria and foreign particles contained in the supernatant were thoroughly eliminated by filtration using a 0.22-µm pore size^17^.

### Gas chromatography time-of-flight mass spectrometry analysis

Frozen EV samples were thawed and prepared to analyze using gas chromatography time-of-flight mass spectrometry. Detailed experimental procedure is described in our previous studies^18,19^.

### DNA extraction and sequencing

Bacterial EVs were boiled using a heat block for 40 min at 100 °C and then the remaining particles and waste were removed by centrifugation at 13,000 rpm for 30 min at 4 °C. The DNA was extracted from supernatants using a DNeasy PowerSoil kit (QIAGEN, Germany). The DNA of bacterial EVs in each sample was quantified by QIAxpert (QIAGEN, Germany). V3-V4 regions of the 16 S rDNA gene was amplified with primers; 16S_V3_F (5′ - TCGTCGGCAGCGTCAGATGTGTATAAGAGACAGCCTACGGGNGGCWGCAG -3′) and 16S_V4_R (5′ - GTCTCGTGGGCTCGGAGATGTGTATAAGAGACAGGACTACHVGGGTATCTAATCC -3′). The library preparation was performed using PCR products and each amplicon was sequenced by MiSeq (Illumina, USA).

### Bioinformatics

Paired-end reads that matched the adapter sequences were trimmed by cutadapt (version 1.1.6)^20^. The resulting FASTQ files containing paired-end reads were merged with CASPER and then quality filtered with Phred (Q) score based criteria described by Bokulich^21,22^. Any read shorter than 350 bp or longer than 550 bp after merging was also discarded. To identify the chimeric sequences, a reference-based chimera was detected by VSEARCH against the SILVA gold database^23^. And then the clustering into Operational Taxonomic Units (OTUs) was conducted using VSEARCH with the *de novo* clustering algorithm under a 97% sequence similarity. Exclusion criteria of OTUs was a containing one read sequence in only a sample. The representative sequences of the OTUs were finally classified using the SILVA 128 database with UCLUST (script on QIIME version 1.9.1)^24^. We applied normalization to the data sets using the total count method as described by Previous study^25^. Chao indices, estimators of taxa richness per individual, were estimated to measure the alpha diversity of each sample. The metagenome biomarkers selection in the diagnostic model was based on the relative abundances at the genus level. The criteria of false discovery rate (FDR)-adjusted p-values as determined by Wilcoxon test, fold-changes and average relative abundances were less than 0.05, greater than 2-fold and greater than 0.5% in any group, respectively. In addition, we selected metabolome biomarkers with adjusted P-values less than 0.05 and changes greater than 2-fold. All diagnostic models were calculated by logistic regression based on Akaike information criteria using stepwise selection method with training and test sets selected randomly at an 80:20 ratio. The performance values, such as AUC, sensitivity, specificity, and accuracy, were reported using validataion set. A logistic regression model was built using individual omics data from metagenomic and metabolic biomarkers; its accuracy was then compared to a combined model of metagenomics and metabolic biomarkers to discriminate cancer from healthy controls.

### Statistics for metabolomics data

Multivariate and univariate analyses were conducted using Metaboanalyst 4.0. Normalized data sets using log transformation and pareto scaling were analyzed and principal component analysis (PCA) was used to examine differentiation in overall metabolic profiles between the groups. Univariate analysis using false discovery rate (FDR)-adjusted P-value was used for the selection of metabolic candidates. Significant differences between the healthy control group and CRC patient group were determined using the Wilcoxon test for continuous variables. Findings were considered significant if the p-value was less than 0.05.

### Statistics for metagenomic data

Alpha diversity of microbial composition for richness and evenness was analyzed using the Chao1 index and Shannon’s index to compare diversity between the healthy control and CRC patient groups. Principal coordinate analysis (PCoA) based on Bray-Curtis similarity for beta diversity was used to visualize relationships between samples. R (version 3.5.1) was used for all statistical analyses.

## Results

### Microbiome analysis of microbe-derived EVs in stool samples from CRC patients and healthy subjects

**Figure 1:**
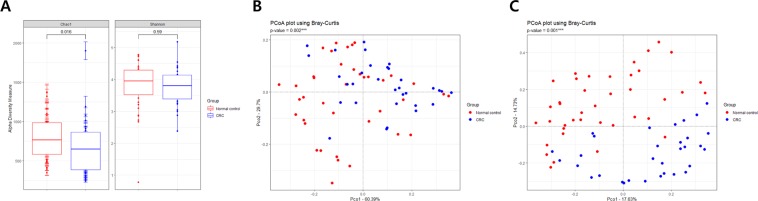
Alpha and beta diversity comparisons of microbiomes collected from CRC patients and healthy controls. Analysis was performed using sequencing data for the 16S rDNA V3 and V4 regions, with a rarefaction depth of 10,000 reads per sample. (**A**) Chao1 and Shannon indexes indicate alpha diversity. Whiskers in the boxplots represent the range of the minimum and maximum alpha diversity values within a population, excluding outliers. Principal coordinate analysis (PCoA) plots represent beta diversity at the (**B**) phylum and (**C**) genus levels. Red circles represent healthy control individuals and blue circles represent CRC patients. CRC, colorectal cancer.

To investigate the microbial compositions of stool EVs from the CRC patients and healthy controls, metagenome analysis was performed based on 16 S rDNA amplicon sequencing. Comparison of alpha diversity in the CRC patients and healthy controls revealed no significant differences based on the Chao1 and Shannon indexes, as shown in Fig. 1(A). Beta diversity at the phylum and genus levels was represented through principal coordinate analysis (PCoA) (Fig. 1(B)). A comparison of beta diversity at the genus level demonstrated a clear separation between the groups compared to the phylum-level clusters.

**Figure 2:**
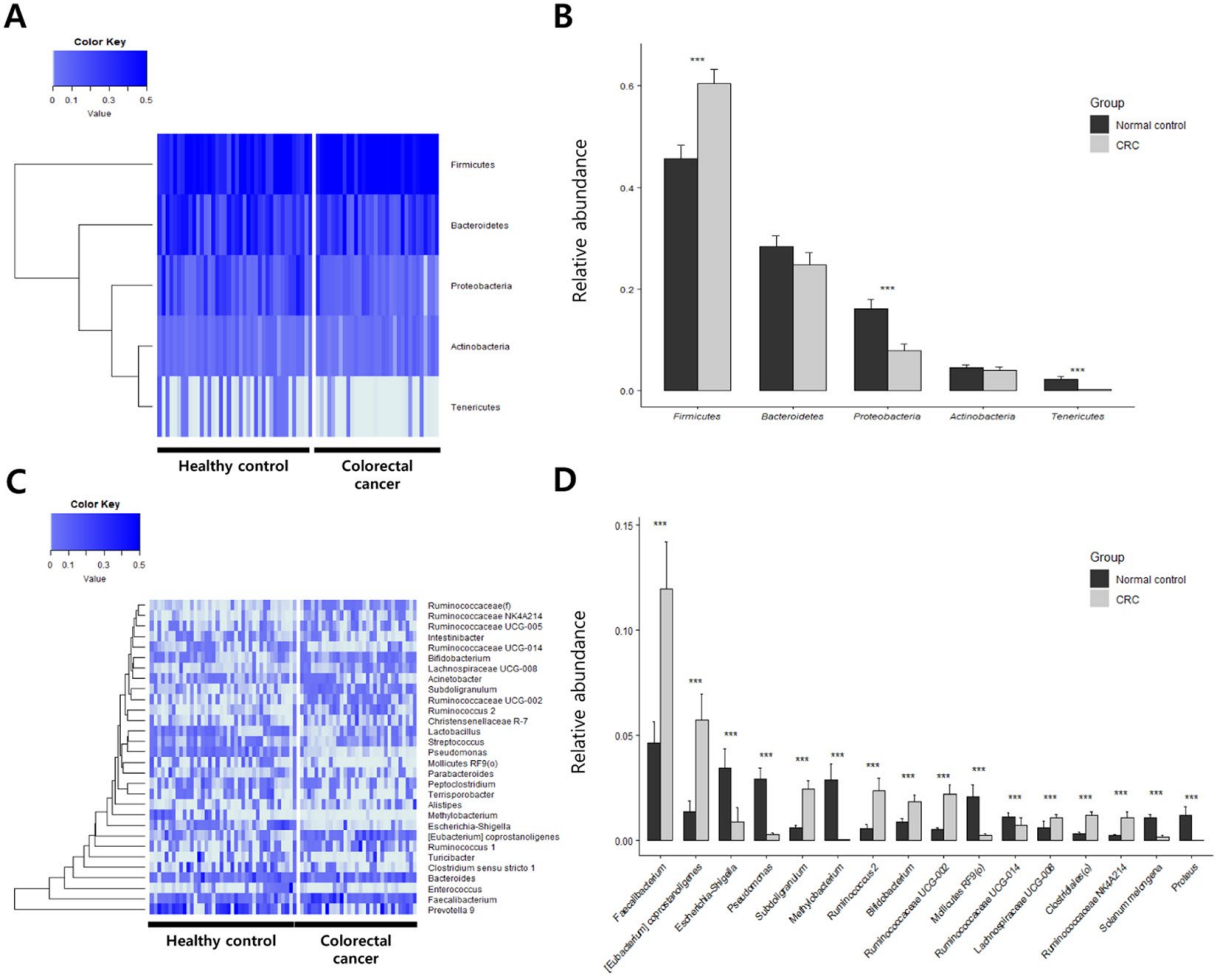
Comparison of bacterial composition of EVs from CRC patients and healthy controls. Heat maps represent the relative abundance of microbes (**A**) at the phylum level and (**C**) at the genus level in CRC patients and healthy controls. Cells with an abundance value close to zero are represented in light blue, and those with an abundance value larger than 0.5 are indicated in dark blue. The total abundance is 1. Bar graphs represent microbial populations that were significantly different in abundance between CRC patients and healthy controls (**B**) at the phylum level and (**D**) at the genus level. The gray-colored bar indicates the relative abundance in CRC patients, and the black-colored bar indicates the relative abundance in healthy controls. **P < 0.05, ***P < 0.01 between CRC patients and healthy controls. EV, extracellular vesicle; CRC, colorectal cancer.

**Figure 3:**
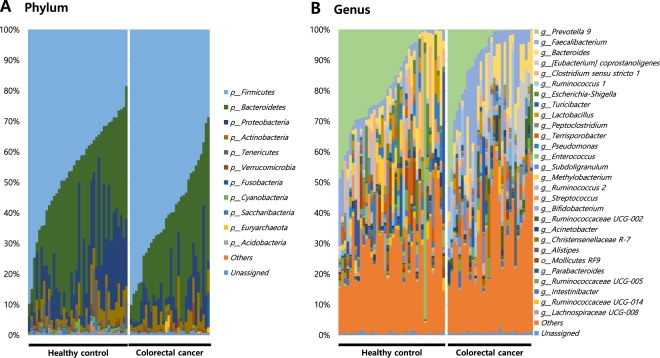
Taxonomic profile of microbe-derived EVs in CRC patients and healthy controls. Analyses were performed for 16S rDNA V3 and V4 regions data, with a rarefaction depth of 10,000 reads per sample. Relative taxon abundance plots for CRC patients and healthy controls at the (**A**) phylum and (**B**) genus levels. Individuals are represented along the horizontal axis and relative taxon frequency is denoted on the vertical axis. EV, extracellular vesicle; CRC, colorectal cancer.

Heat maps further allowed for the visualization of relative changes in microbial abundance at the phylum and genus levels (Fig. 2(A,C)). In the comparison of phylum levels, three individual phyla were found; *Firmicutes* was significantly increased in CRC patients, whereas the level of *Proteobacteria* and *Tenericutes *was decreased (Fig. 2(B)). The bar graphs in Fig. 2(D) demonstrate that the microbial compositions changed at the genus level in CRC patients compared to those in healthy controls. Detailed records of those data are listed in Table 1. There was a significant difference observed in 34 bacterial genera between the CRC group and the healthy control group. As presented in Table 1, the proportions of *Actinomyces*, *Rothia*, *Propionibacterium*, *Bacteroidiales S24-7 group, Chloroplast*, *Lachnospiraceae NK4A136 group*, *Ruminococcaceae UCG-014, Staphylococcus,Methylobacterium*, *Solanum melongena*, *Sphingomonas*, *Escherichia-shigella*, *Proteus*, *Pseudomonas*, *Saccaribacteria*, and *Mollicutes* were decreased in the CRC patients compared to those in the healthy controls (P < 0.05), whereas the proportions of *Bifidobacterium*, *Collinsella*, *Blautia*, *Lachnoclostridium*, *Lachnospiraceae UCG-008, Dorea, Eubacterium coprostanoligenes group*, *Ruminococcus 2, Faecalibacterium*, *Ruminococcaceae NK4A214*, *Ruminococcaceae UCG-002*, *Ruminococcus*, *Subdoligranulum*, *Ruminococcaceae*, *Catenibacterium*, *Parvimonas, Ruminiclostridium 5, Enterobacter*, and *Diaphorobacter* were significantly enriched (P < 0.05). The predominant observation regarding these changes was that microbial compositions of *Proteobacteria* were larger while the compositions of *Firmicutes* were reduced, except for those of *Lachnospiraceae UCG-008*, *Ruminococcaceae UCG-014*, and *Staphylococcus*. Moreover, among *Proteobacteria*, *Proteus* spp. were dramatically altered in the CRC patients and was absent in healthy controls. Taxonomic profiles are presented in Fig. 3.

**Table 1:** List of microbiomes significantly changed in CRC.

Phylum	Genus	MAV of healthy controls (%)	MAV of CRC patients (%)	Log2	p-value	FDR adjusted p-value	Regulation
				(Fold change)	(Wilcoxon)	(Wilcoxon)	
Actinobacteria	Actinomyces	0.646	0.053	−3.613	0.000	0.001	Down
	Bifidobacterium	0.871	1.832	1.073	0.001	0.014	Up
	Rothia	0.533	0.016	−5.099	0.000	0.000	Down
	Propionibacterium	0.685	0.164	−2.061	0.000	0.000	Down
	Collinsella	0.065	0.953	3.880	0.000	0.000	Up
Bacteroidetes	Bacteroidales S24-7 group (f)	0.610	0.491	−0.313	0.003	0.031	Down
Cyanobacteria	Chloroplast (o)	0.507	0.059	−3.104	0.000	0.000	Down
Firmicutes	Blautia	0.187	0.599	1.677	0.000	0.000	Up
	Lachnoclostridium	0.096	0.740	2.943	0.000	0.000	Up
	Lachnospiraceae NK4A136 group	0.561	0.170	−1.718	0.002	0.019	Down
	Lachnospiraceae UCG-008	0.591	1.069	0.855	0.000	0.000	Up
	Dorea	0.450	0.510	0.181	0.000	0.008	Up
	[Eubacterium] coprostanoligenes group	1.332	5.696	2.096	0.000	0.000	Up
	Ruminococcaceae UCG-002	0.484	2.180	2.171	0.000	0.002	Up
	Ruminococcus 2	0.530	2.329	2.136	0.000	0.002	Up
	Subdoligranulum	0.562	2.408	2.099	0.000	0.000	Up
	Ruminococcaceae (f)	0.317	1.187	1.905	0.000	0.000	Up
	Ruminococcaceae UCG-014	1.098	0.706	−0.638	0.000	0.004	Down
	Faecalibacterium	4.624	11.974	1.373	0.000	0.003	Up
	Ruminococcaceae NK4A214 group	0.209	1.045	2.319	0.001	0.009	Up
	Staphylococcus	0.819	0.410	−0.998	0.003	0.033	Down
	Catenibacterium	0.041	0.729	4.161	0.000	0.000	Up
	Parvimonas	0.028	0.812	4.840	0.001	0.013	Up
	Ruminiclostridium 5	0.083	0.527	2.672	0.001	0.010	Up
Proteobacteria	Methylobacterium	2.846	0.027	−6.743	0.000	0.007	Down
	Solanum melongena (eggplant)	1.046	0.141	−2.887	0.000	0.000	Down
	Sphingomonas	0.548	0.172	−1.674	0.000	0.000	Down
	Diaphorobacter	0.000	0.974	12.397	0.000	0.001	Up
	Escherichia-Shigella	3.427	0.871	−1.975	0.000	0.000	Down
	Proteus	1.169	0.000	«	0.000	0.000	Down
	Pseudomonas	2.913	0.242	−3.589	0.000	0.000	Down
	Enterobacter	0.158	0.816	2.370	0.004	0.035	Up
Saccharibacteria	Saccaribacteria (p)	0.540	0.132	−2.029	0.001	0.017	Down
Tenericutes	Mollicutes RF9 (o)	2.053	0.208	−3.304	0.001	0.011	Down

MAV indicates mean abuandance value; Fold-change indicates the difference in the mean abundance values between CRC patients and healthy controls; for comparison of the relative abundance of the two groups, false discovery rate-adjusted p-values were calculated.

### Metabolic profiling of stool EVs from CRC patients and healthy subjects

**Figure 4:**
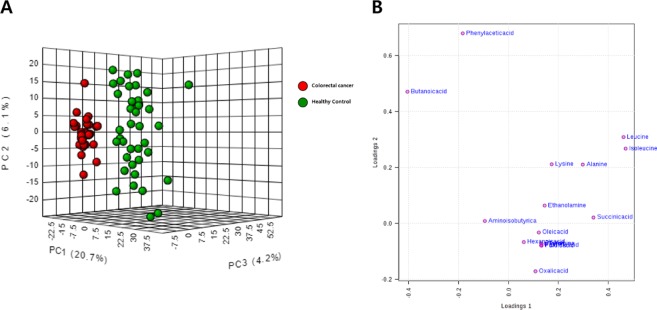
Distinct metabolic profiling between CRC patients and healthy controls. Metabolic profiling was performed using GC-TOF-MS. (**A**) Score plot of the three-dimensional principal component analysis (PCA) shows metabolic patterns of stool EV samples. Red circles indicate colorectal cancer and green circles indicate healthy controls. (**B**) Loading plots of PC1 and PC2 from the PCA results of differentially accumulating metabolites from CRC patients versus healthy controls. EV, extracellular vesicle; CRC, colorectal cancer; PC, principal component.

To assess the profile of small-molecule metabolites in EVs, we conducted a global metabolomics analysis using GC-TOF-MS. In three-dimensional PCA score plots, as shown in Fig. 4(A), three PCs (PC1–3) clearly separated the metabolomics profiles of healthy controls and CRC patients. The metabolites identified by multivariate analysis were selected according to their Q-values, which are P-values adjusted for the FDR. The metabolites that showed statistical significance (Q < 0.05) are listed in Table 2 and Table S2. The loading plot in Fig. 4(B) shows the metabolites that effectively differentiated CRC patients from healthy controls. The most frequent small-molecule metabolites were classified as amino acids that were more abundant in CRC patients. Furthermore, metabolites with alcohol forms (ethanolamine and phenol), carboxylic acids (furoic acid, succinic acid, and oxalic acid), and fatty acids (hexanoic acid, palmitic acid, and oleic acid) were enhanced in CRC patients compared to those in healthy controls. Notably, bacterial metabolites such as aminoisobutyric acid and butanoic acid were reduced.

**Table 2:** List of metabolites significantly changed in CRC.

Metabolite	Class	Fold-change	p-value	Regulation
Leucine	Amino acid	2.433	5.75E-03	Up
Isoleucine		2.192	5.23E-03	Up
Alanine		1.667	4.31E-02	Up
Lysine		1.441	1.48E-02	Up
Tyramine		1.423	1.57E-22	Up
Aminoisobutyric acid		−1.116	6.66E-03	Down
Ethanolamine	Amino alcohol	1.384	4.56E-04	Up
Phenol	Aromatic alcohol	1.589	1.99E-12	Up
Furoic acid	Carboxylic acid	1.417	1.31E-29	Up
Succinic acid		3.154	4.46E-09	Up
Oxalic acid		1.396	1.66E-03	Up
Butanoic acid	Fatty acid	−1.300	2.26E-04	Down
Hexanoic acid		1.302	9.38E-06	Up
Palmitic acid		1.436	4.37E-25	Up
Oleic acid		1.317	3.09E-04	Up

Fold-change indicates the difference in the mean abundance values between CRC patients and healthy controls; for comparison of the relative intensities of the two groups, false discovery rate-adjusted p-values were calculated.

### Correlation between microbiome and metabolic profiles in stool EVs

**Figure 5:**
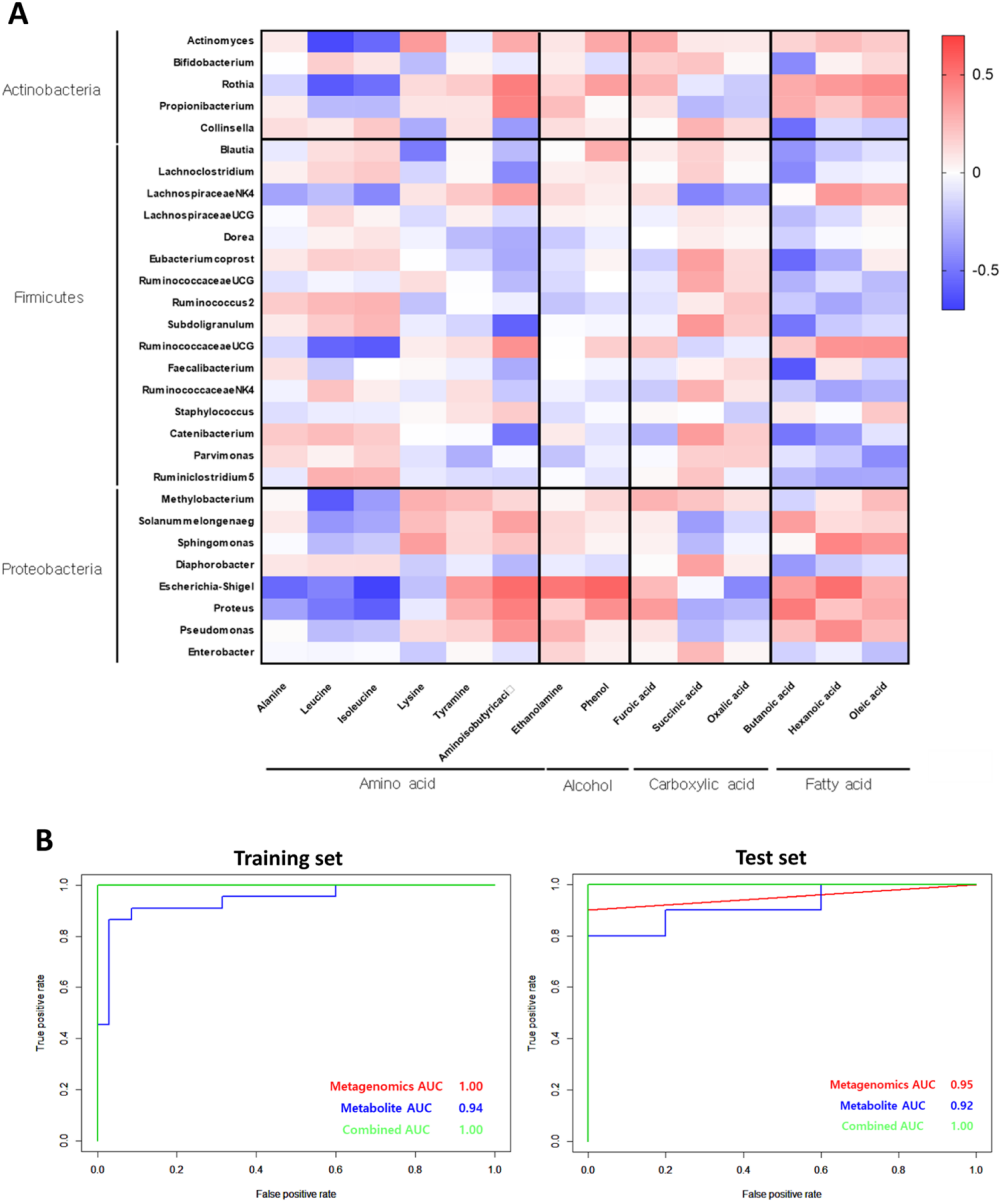
Pearson correlation analysis and the power of the relative abundance of microbiomes and metabolites to discriminate CRC from healthy controls. (**A**) Pearson correlation analysis was performed to investigate the association between metagenomic and metabolomic analysis data. The y-axis represents the result of the metagenomic analysis, and the x-axis represents metabolic biomarkers, both obtained through statistical comparisons. Each square shows the correlation coefficient value. Red squares indicate a positive correlation and blue squares indicate a negative correlation between microbial and metabolite abundances. (**B**) Receiver operating characteristic (ROC) curves for the markers leucine, butanoic acid, isoleucine, succinic acid, *Bifidobacterium*, *Lactobacillus*, *Faecalibacterium*, *Pseudomonas*, and *Methylobacterium* based on their ability to discriminate CRC patients from healthy controls. The red line indicates the model based on metagenomics analysis, the blue line indicates the model based on metabolomics analysis, and the green line indicates the model based on combination of metabolomics and metagenomics data. CRC, colorectal cancer.

A Pearson rank correlation analysis demonstrated a close correlation between the gut microbiota and certain metabolic products (Fig. 5(A)). The relative abundance of most metabolic markers was highly positively correlated with the *Firmicutes* genera. Specifically, several amino acids were enriched according to the consistent regulation of gut flora in CRC patients. These bacteria shared a significant relationship with tyramine, phenol, and hexanoic acid (r > |0.5|, P < 0.05). Observations for *Proteobacteria* were opposite to those for *Firmicutes*, wherein the *Proteobacteria* family was negatively correlated with these metabolic biomarkers (r < |0.5|, P < 0.05). Among the metabolic biomarkers, carboxylic acids (such as furoic acid, succinic acid, and oxalic acid) and long chain fatty acids (such as palmitic acid and oleic acid) moderately correlated with levels of the entire gut flora.

### CRC diagnostic models based on microbiome and metabolic profiles in stool EVs

To further define the useful biomarkers from the metagenomic and metabolomic biomarkers, a binary logistic regression analysis and an optimized algorithm of the forward stepwise method were employed to construct the best model using these retained biomarkers to distinguish CRC-positive individuals from healthy controls. Ultimately, two metabolites (leucine and oxalic acid) and two bacterial genera (*Collinsella* and *Solanum melongena*) were selected. Figure 5(B) shows the receiver operation curve of the logistic regression model to discriminate CRC-positive samples from healthy controls. Using the two metabolic biomarkers, the predictability of CRC was 92.0%with 80.0% sensitivity and 100% specificity. The two selected metagenomics biomarkers resulted AUC value (95.0%) with 90.0% sensitivity and 100% specificity. Each AUC values were slightly lower in test set compared to training set. Integration of these two panels of omics data led to an AUC of 100% with relevant accuracy in discriminating between CRC-positive samples and healthy controls (Figure 5(B) and Table S3). A permutation test of the logistic regression model was conducted for assessment and to exclude over-fitting (Table S4). Although the patients were diagnosed more accurately in the combined model, metagenomic biomarkers were found to fit on this model more efficiently compared to metabolic biomarkers (Table S4). These data suggest that these potential representative markers of CRC, a combination of metagenomic and metabolomic biomarkers, might diagnose CRC more accurately than a single omics biomarker.

## Discussion

In the present study, we performed metabolic analysis and microbiome profiling of EVs obtained from stools of CRC patients and healthy volunteers to identify metabolites that change with pathophysiology and to suggest possible correlations with gut microbes, respectively. Through 16 S rDNA sequencing, we found compositional changes in bacteria belonging to the *Firmicutes* and *Proteobacteria* phyla in CRC patients compared to that in healthy controls. Based on global metabolomics profiling, several amino acids and carboxylic acids were more abundant in the presence of cancer, whereas some microbe-associated metabolites such as aminoisobutyric acid and butanoic acid were less abundant. To the best of our knowledge, this is the first study to report the association of CRC development with the microbiome and metabolomics using stool EVs.

Accumulating studies show that several bacterial species seem to be involved in the pathogenesis of CRC. For instance, *Streptococcus bovis* is predominant in patients with colon cancer, which colonize approximately 20–50% of the gut but less than 5% in healthy individuals^26^. Elevation of the *Bacteroides* and *Prevotella* population is also an indicative marker of CRC based on metagenome analysis^27^. In the present study, we observed dynamic changes in the *Firmicutes* and *Proteobacteria* phyla from the EVs of CRC patients compared to those in healthy controls. A higher abundance of *Firmicutes* and *Fusobacteria* has been primarily reported, whereas *Proteobacteria* were less abundant in individuals with CRC^27^. *Firmicutes* including taxa such as *Eubacterium*, *Clostridium*, *Lactobacillus*, and *Peptostreptococcaceae*, have been shown to be involved in energy resorption^28^. This might depend on the bacterium’s ability to rapidly exploit unabsorbed, labile amino acids and peptides from the diet. Most of these organisms have proteolytic activity, thereby degrading recalcitrant proteins that have relatively long transit times in the gut^28,29^. In this study, we demonstrated that several genera of *Firmicutes* were increased in CRC patients, such as *Eubacterium*, *Faecalibacterium*, those of the *Ruminococcaceae* family, and *Catenibacterium*. However, there were some exceptional cases wherein *Lactobacillus* and *Clostridium* were reduced compared to levels in healthy controls.

*Firmicutes* and *Bacteroidetes* are the dominant phyla in the gut microbial community, whereas other phyla such as *Proteobacteria*, *Actinobacteria*, and *Verrucomicrobia*, are generally less abundant. Compositional changes in the phyla, such as increased prevalence of *Proteobacteria*, can be easily influenced by inflammation of the gastrointestinal tract. An increased proportion of *Proteobacteria* including the families *Enterobacteriaceae*, *Pasteurellaceae*, and *Neisseriaceae* distinguishes a Crohn’s disease-related bacterial community from that of healthy subjects^30^. One possible explanation for the richness of *Proteobacteria* is that whereas the mucosal immune system is obligated to clear pathogens, an inappropriate immune response abolishes the homeostasis of the gut flora, leading to dysbiosis and triggering local and systemic inflammation and malfunction of the endogenous metabolism of the host^7,29,31^. The potential distinct functions of *Proteobacteria* in colon tumors are still unclear, although they are known as commensal bacteria that possess potential pathogenic features. Here, we suggest possible influential factors that might contribute to their functional repertoire, including toxic byproducts, virulence factors, and other parameters that propagate interactions between the bacteria and their gut environment, rather than acute and chronic inflammation.

Enhanced amino acid levels are closely related to CRC risk. Possible reasons for this might include the following: changes in dietary habits, as high protein intake has long been regarded the most important lifestyle risk factor for colorectal cancer^32^; inflammation, which diminishes the absorption of nutrients in patients with cancer^5,27^; degradation of dietary protein by fermenting bacteria in the distal colon of patients with CRC, which elevates the levels of amino acid metabolites in stool^16^. One study on amino acid utilization and catabolism in bacteria from the human intestine identified bacteria belonging to the *Clostridium* clusters (*Bacillus*, *Lactobacillus* and some *Proteobacteria*) as those mostly responsible for the fermentation of amino acids^29^. These organisms can utilize amino acids in the gut, such as lysine, proline, phenylalanine, and tryptophan, to produce small molecules including ammonia, hydrogen sulfide, nitric oxide, polyamines, and alcoholic compounds^33,34^. As mentioned earlier, *Firmicutes* can catalyze amino acids for energy recycling, whereas *Proteobacteria* can degrade amino acids including undigested proteins. This dysbiosis preferentially affects amino acid metabolism. Short-chain fatty acids (SCFAs) including butanoic acid and aminoisobutyric acid are a well-established energy source in the human intestine. These microbial metabolites play a role in modulating host metabolic and immune responses^35^. The current study demonstrated that diet-related SCFAs prevent disease and provide therapeutic implications for CRC^36^, as these bacterial metabolites are restricted to CRC patients and not present in healthy controls.

In the present study, we observed EVs with metagenomic profiles similar to those from previous studies utilizing stool-based metagenome analyses of CRC patients. However, alterations in microbial compositions based on EVs do not directly reflect the proportional changes of gut microbes. Bacteria secrete small vesicles of various forms, such as OMVs, EVs, and ectosomes, to transfer cellular components and modulate signaling pathways. These vesicles have become promising research tools to discover therapeutic targets, develop drug delivery systems, and quantify microbial compositions by utilizing their properties^8,37^. Thus, systematic and comprehensive studies integrating multiple sources are required to understand the complexity of EV-triggered intercellular and interkingdom communication. Global metabolomic analyses, based on the technique of ultra-high performance gas chromatography, have been established to profile a broad range of metabolites existing in the EVs, enabling the identification of cooperation between microbiomes and cancer development. These findings based on stool-based analysis require confirmation, and further functional studies are needed to determine whether the bacteria influence cancer development.

This study is the first to demonstrate the correlation between microbial changes and metabolic alternations within EV samples from patients with CRC. There was a strong association between the abundance of gut flora (*Firmicutes* and *Proteobacteria*) and relevant candidate metabolites (predominantly amino acids). This suggests that the altered composition of macronutrient-fermenting and degrading bacteria in CRC might result in the accumulation of amino acids and the depletion of energy sources. Moreover, our findings indicate that EVs secreted by gut microbes carry a dynamic range of metabolic information reflecting the host’s nutritional state, metabolism, and immune responses in the presence of disease.

## Supplementary information

Supplementary tables.

Supplementary data.

## Data Availability

The raw sequence data and processed data of metagenome analysis are available through the Sequence Read Archive under BioProject ID: PRJNA601555. The metabolomics data are available in the electronic Supplementary Material and at the NIH Common Fund's National Metabolomics Data Repository (NMDR) website [Project ID: PR000888].
