# Supplementary material for: Colorectal cancer diagnostic model utilizing metagenomic and metabolomic data of stool microbial extracellular vesicles
Source: Sci Rep. 2020 Feb 18;10:2860. doi: 10.1038/s41598-020-59529-8 (PMC7029032; doi:10.1038/s41598-020-59529-8)
Supplement: Supplementary file 1 — Supplementary tables. [file 41598_2020_59529_MOESM1_ESM.docx]

*Supplementary tables*

**Colorectal cancer diagnostic model utilizing metagenomic and metabolomic data of stool microbial extracellular vesicles**

Da Jung Kim^1, 7*^, Jinho Yang^2,6*^, Hochan Seo^2^, Won Hee Lee^2^, Dong Ho Lee^3^, Sungmin Kym^4^, Young Soo Park^3^, Jae Gyu Kim^5^, In-Jin Jang^1^, Yoon-Keun Kim^2**^ and Joo-Youn Cho^1, 7**^

^1^Department of Clinical Pharmacology and Therapeutics, Seoul National University College of Medicine and Hospital, 101 Daehak‑ro, Jongno‑gu, Seoul 03080, Republic of Korea

^2^Institute of MD Healthcare Inc, Seoul, Korea

^3^Department of Internal Medicine, Seoul National University Bundang Hospital, Gyeonggi-do, Republic of Korea

^4^Department of Internal Medicine, Inje University Haeundae Paik Hospital, Inje University College of Medicine, Busan, Republic of Korea

^5^Department of Internal Medicine, Chung-Ang University College of Medicine, Seoul, Republic of Korea

^6^Department of Health and Safety Convergence Science, Korea University, Seoul, Republic of Korea

^7^Department of Biomedical Sciences, Seoul National University College of Medicine, 101 Daehak‑ro, Jongno‑gu, Seoul 03080, Republic of Korea

*These authors equally contributed to this work.

**These co-corresponding authors equally contributed to this work.

**Supplementary Table 1.** Characteristics of the study population

|  | | **Healthy control** | **Colorectal cancer** | **p-value** |
| --- | --- | --- | --- | --- |
| **Total No.** | | 40 | 32 |  |
| Sex | Male | 22 | 20 | 0.6885 |
|  | Female | 18 | 12 |  |
| Stage | Stage 0 | - | 1 |  |
|  | Stage Ⅰ | - | 7 |  |
|  | Stage Ⅱ | - | 12 |  |
|  | Stage Ⅲ | - | 9 |  |
|  | Stage Ⅳ | - | 3 |  |
| Tumor location | Cecum | - | 2 |  |
|  | Ascending | - | 6 |  |
|  | Transverse | - | 1 |  |
|  | Sigmoid | - | 12 |  |
|  | Rectum | - | 7 |  |
| **Age** | Mean ± SD | 63.3 ± 9.2 | 63.6 ± 9.9 | 0.8788 |
|  | Range (Median) | 49 ~ 78 (64.5) | 45 ~ 80 (64.0) |  |
| **CEA** | Mean ± SD (ng/ml) | - | 3.21 ± 4.83 |  |
|  | No. (0 ~ 5 ng/ml) | - | 29 |  |
|  | No. (5 ng/ml <) | - | 2 |  |

**Supplementary Table 2.** List of metabolites observed via GC-TOFMS

| **tR (sec)** | **Unique mass (m/z)** | **Exact mass molecule** | **Similarity** | **Reverse** | **Probability** | **Chemical Formula** | **Identity** | **Level of MSI** |
| --- | --- | --- | --- | --- | --- | --- | --- | --- |
| 315.15 | 86 | 131.1729 | 777 | 845 | 6243 | C6H13NO2 | Leucine | 2 |
| 325.2 | 86 | 131.1729 | 737 | 739 | 6213 | C6H13NO2 | Isoleucine | 2 |
| 291.15 | 116 | 89.0932 | 848 | 848 | 6235 | C3H7NO2 | Alanine | 2 |
| 599.65 | 174 | 146.19 | 712 | 717 | 6382 | C6H14N2O2 | Lysine | 2 |
| 745.25 | 174 | 137.179 | 877 | 889 | 4563 | C8H11NO | Tyramine | 2 |
| 444.25 | 174 | 103.1198 | 834 | 845 | 6732 | C4H9NO2 | Aminoisobutyric acid | 2 |
| 365.25 | 174 | 61.0831 | 742 | 866 | 4353 | C2H7NO | Ethanolamine | 2 |
| 953.35 | 441 | 94.1112 | 774 | 774 | 9579 | C6H6O | Phenol | 2 |
| 306.2 | 189 | 112.0835 | 737 | 858 | 6264 | C5H4O3 | Furoic acid | 2 |
| 453.35 | 233 | 118.088 | 876 | 896 | 4964 | C4H6O4 | Succinic acid | 2 |
| 506.55 | 116 | 90.0349 | 709 | 854 | 4350 | C2H2O4 | Oxalic acid | 2 |
| 320 | 145 | 88.1051 | 851 | 870 | 3894 | C4H8O2 | Butanoic acid | 2 |
| 275.8 | 173 | 116.1583 | 861 | 878 | 9200 | C6H12O2 | Hexanoic acid | 2 |
| 631.8 | 117 | 256.4241 | 899 | 899 | 6134 | C16H32O2 | Palmitic acid | 2 |
| 679.35 | 129 | 282.4614 | 723 | 725 | 6091 | C18H34O2 | Oleic acid | 2 |

**Supplementary Table 3.** Performance values of the logistic regression model using test set

| **Prediction** | **Negative** | | | **Positive** | | |
| --- | --- | --- | --- | --- | --- | --- |
|  | Metabolomics | Metagenomics | Metabolomics+ Metagenomics | Metabolomics | Metagenomics | Metabolomics+ Metagenomics |
| **Negative** | 5 | 5 | 5 | 2 | 1 | 0 |
| **Positive** | 0 | 0 | 0 | 8 | 9 | 10 |

**Supplementary Table 4.** Results of permutation test

|  | Markers | Parameter | p-value | Training set | | | | Test set | | | |
| --- | --- | --- | --- | --- | --- | --- | --- | --- | --- | --- | --- |
|  |  |  |  | Sen | Spe | Acc | AUC | Sen | Spe | Acc | AUC |
| Metabolomics | Leucine | 0.0000005 | 0.000 | 0.86 | 0.94 | 0.91 | 0.94  (CI: 0.92 - 0.96) | 0.80 | 1.00 | 0.87 | 0.92  (CI: 0.84 - 1.00) |
|  | Oxalic acid | 0.0000047 | 0.000 |  |  |  |  |  |  |  |  |
| Metagenomics | Collinsella | 45500 | 0.000 | 1.00 | 1.00 | 1.00 | 1.00  (CI: 0.98 - 1.00) | 0.90 | 1.00 | 0.93 | 0.95  (CI: 0.88 - 1.00) |
|  | Solanum melongena | -81220 | 0.000 |  |  |  |  |  |  |  |  |
| Metabolomics+ Metagenomics | Leucine | 0.000004 | 1.000 | 1.00 | 1.00 | 1.00 | 1.00  (CI: 0.98 - 1.00) | 1.00 | 1.00 | 1.00 | 1.00  (CI: 0.93 - 1.00) |
|  | Oxalic acid | -0.000013 | 1.000 |  |  |  |  |  |  |  |  |
|  | Collinsella | 52930 | 0.000 |  |  |  |  |  |  |  |  |
|  | Solanum melongena | -17420 | 0.000 |  |  |  |  |  |  |  |  |

Sen: Sensitivity, Spe: Specificity, Acc: Accuracy, AUC: Area Under Curve
